# Investigation of the Protective Function of a Lignin Coating of Natural Fiber Geotextiles against Biodegradation

**DOI:** 10.3390/ma16134849

**Published:** 2023-07-06

**Authors:** Cigdem Kaya, Thomas Stegmaier, Götz T. Gresser

**Affiliations:** Deutsche Institute für Textil- und Faserforschung, Körschtalstraße 26, 73770 Denkendorf, Germany; thomas.stegmaier@ditf.de (T.S.);

**Keywords:** textiles, geotextiles, natural fibers, coating, lignin, biodegradation, soil burial test

## Abstract

Natural fibers do not have a long life in soil; therefore, they cannot replace synthetic textiles in many applications. However, in order to solve ever-increasing global environmental problems due to microplastics, more and more natural polymers must be used, creating a need for research into the sustainable life extension of natural fibers. Lignin is, along with cellulose, a main component of wood, and is produced in large quantities as waste during paper production. With appropriate processing, lignin can be exploited/used as a textile auxiliary to combine the strength-enhancing properties of textiles made from natural fibers with the protective properties of a lignin coating. However, there is not yet sufficient research on how to integrate lignin into textile applications. For this purpose, in this study, we have investigated whether thermoplastic lignin can be processed as a surface protective coating. We tested lignin as a yarn coating to extend the service life of cellulosic textiles. Cotton yarns have been coated with lignin in variations of coating mass, characterized and investigated by means of soil burial tests. As the soil burial tests conducted in climate chamber and outdoor field environments showed, the lifespan of textiles made from natural fibers can be significantly extended with a lignin coating. Long-term resilience has been demonstrated in standard burial tests. In the outdoor tests, the lignin coating was still fully intact, even after about 160 days of burial. The textile materials coated in this way enable sustainable applications, especially for geotextiles. They have an adjustable, sufficiently long service life; however, they are still biodegradable, and can therefore replace some applications, such as vegetating trench/brook slopes, with synthetic materials. Lignin-coated textiles have the potential to significantly reduce the carbon footprint, reduce not only the dependence on petroleum-based products but also the amount of microplastics entering the environment. Further research can be conducted to improve lignin compounding in terms of other interesting properties for specific textile applications. Process optimization could increase the protective effect and further extend the life of useful textiles in soil.

## 1. Introduction

The use of technical textiles in/on soil, also known as geotextiles, is a rapidly growing market. Geotextiles are mostly permeable textile materials which are mainly used for filtration, drainage, separation, reinforcement, and stabilization purposes. To date, fibers made from oil-based polymers (polypropylene, polyethylene and polyester), which have an extremely long service life, have primarily been used. The majority of geotextiles remain in the ground—even if their function is no longer required—and thus pollute our environment with non-degradable plastics. For this purpose, new solutions from manufacturers as well as users based on renewable raw materials, which are degradable in the soil after a certain time, are required. However, the geotextiles made from natural fibers that have been available to date do not meet the requirement for a sufficient service life for many applications.

Natural fibers consist mainly of high molecular weight carbohydrate polymers, cellulose, or proteins. Microorganisms degrade the lignocellulose present in natural fibers. They can attack the carbohydrate polymers in the cell wall, resulting in a loss of strength.

Lignocellulose also undergoes photochemical degradation when exposed to ultraviolet light. Natural fibers, therefore, have low biological stability and tend to degrade under conditions favorable for the development of microbial cultures (high moisture and temperature, contact with soil microflora, etc.) that decompose during use. Therefore, it is an extremely important and challenging task to improve the biological protection of cellulosic textile materials.

Galashina et al. refer to studies in which the biostability of cellulosic materials varies according to the content of cellulosic components in the fibre. This content determines the probability or success of microbiological attack. According to this, flax fibres have increased microbiological stability due to their content of lignin, waxy substances and a considerable number of microelements, such as heavy metals and rare earth metals. In contrast, pectin substances, hemicelluloses and active ingredients on the fibrous materials (e.g. starch) during the treatment process are a growth medium for microbial cultures. Easily hydrolysable polysaccharides favour the degradation process of cotton and flax. For this reason, products made from "raw" fibres can be more easily degraded compared to fibres that have been cleaned of foreign and natural impurities [1].

Arshad et al. investigated the biodegradation of fabric samples of natural fibers (cotton, jute, linen, wool) under the attack of microorganisms present in the soil, using a standard soil burial method in which textile materials were directly buried and an indirect method (non-standard method) in which textile materials were filled in hydrophilic and hydrophobic bags and thus exposed to the soil material. Visual analysis and microscopic methods show that the biodegradation of cellulosic fibers proceeds in a similar manner. The only difference is the timing of biodegradation. The fastest biodegradation effects were observed in the linen fabric because the fibers in the yarns were not tightly twisted, resulting in the better accessibility of the material to the microorganisms in the soil. The lowest biodegradation occurred when flax/PET-blended fabric was exposed to soil conditions, which again related to the structure of the material: of all the cellulosic materials, the mass per unit area of this fabric was the highest, and the polyester fibers had no effect on degradation. Microscopic observations, FTIR, and TGA analyses showed that most of the cellulose was degraded by the microorganisms, while the PET fibers remained undamaged. Wool is more resistant to attack by microorganisms due to its molecular structure and surface area. These two factors make it difficult for microorganisms present in the soil to penetrate the structure of the wool and biodegrade it [2].

Warnock et al. published a field study to determine the biodegradation rates of 100% rayon, cotton, and Tencel^®^ fabrics buried in an aerobic *Captina* silt loam soil (fine muddy, siliceous, active). At an optimal soil moisture and a soil temperature of approximately 25 °C, during the field study, rapid biodegradation of cellulose was expected. The amount of material remaining over time varied: the fabric materials showed a rapid biodegradation of rayon, intermediate biodegradation of biodegradation of cotton, and slow biodegradation of Tencel^®^. The biodegradation of the samples could be described by linear kinetics. The rate constants were significantly different and followed the decreasing order of rayon > cotton > Tencel^®^. The calculated half-lives for rayon, cotton, and Tencel^®^ were 22, 40, and 94 days, respectively. With an increasing amorphous region in the structure and decreasing length of the polymer chains, the availability of the cellulose substrate for microbial metabolism increased and resulted in faster biodegradation of the fabric sample [3].

In another study, the biodegradation of fabrics made from fibers of cotton, viscose, modal, Tencel^®^, polylactic acid (PLA), polyethylene terephthalate (PET), and polyacrylonitrile (PAN) under the attack of microorganisms was investigated using the soil burial method for two different time intervals (1 month and 4 months). The study showed that, even after 1 month, the cellulose fabric samples changed both physically and chemically. Among the cellulosic fibers, weight loss for modal, cotton, and viscose fabrics was 90% and showed high degradation, whereas Tencel^®^ fibers showed the lowest value of 60% at a 4-month burial interval. Within the synthetic fabrics, only the PLA fabric lost weight. Higher levels of moisture and water absorption, i.e., higher hydrophilicity, lead to greater biodegradability. Modal, viscose, cotton, and Tencel^®^ fibers have structures of cellulose due to the different crystallinities, polymer chains, and hygroscopic behavior, which may be the main reasons for the different behaviors in biodegradation [4]. Modal is a type of rayon, a semisynthetic cellulose fiber obtained through the spinning of regenerated cellulose. Therefore, the result is consistent with the study of Warnock et al., who expressed the degradation rate constants as rayon>cotton>Tencel^®^, as described.

Various methods of extending the lifespan of natural fibers in the soil have been investigated. Shaker et al. investigated the biofunctionality of flax fiber-reinforced composites with ZnO nanoparticles. Bioactivity was tested for antibacterial activity (Hemmhof) [5]. The ZnO nanoparticles showed increased bioactivity against microorganisms [6]. The resulting composite was effective and bioactive even at the lowest amount (0.02%) of ZnO nanoparticles, reducing the risk of the biodegradation of the fibers. Galashina et al. immobilized silver nanoparticles in flax fiber composites for protection against microorganisms. The silver nanoparticles have antimicrobial activity, and the produced bioprotected composites are believed to have a lower risk of fiber degradation and longer service life by limiting bacterial growth [1].

Esterification via the acetylation of natural fibers is another method used for the plasticization of natural cellulose fibers, which is mainly used to stabilize the cell wall of wood pulps against moisture and environmental degradation, as well as to improve dimensional stability [7].

Frisoni et al. reported a milder acetylation process. Steam-exploded flax (*Linum usitatissimum*) fibers were heterogeneously acetylated using acetic anhydride and sulfuric acid as a catalyst. After a 13-day incubation, the cellulolytic bacterial strains used in this work degraded unmodified fibers to 25–35% of their initial weight. None of the acetylated fibers studied degraded as rapidly. The extent of biodegradation decreases as the degree of acetylation increases. The bacteria used in this work appear to go to the unmodified cellulose whenever possible [8]. 

Modelli et al. also investigated the extent and rate of the degradation of flax (*Linum usitatissimum*) fibers both in the native state and after the chemical modification of the surface (acetylation or polyethylene glycol (PEG) grafting) under laboratory conditions and in two different biodegradable environments. The degradation of the fibers under aerobic conditions through the action of the microorganisms present in the soil was evaluated by monitoring the carbon dioxide evolution using the ASTM 5988-96 method. In vitro biodegradation experiments were performed by exposing the fibers to a pure culture of CellVibrio fibrovorans bacteria and measuring mass loss as a function of time. The degradation rate of acetylated fibers in soil is nearly equal to that of unmodified fibers, whereas acetylated fibers in a pure culture biodegrade more slowly than native fibers. The opposite is true for PEG-grafted fibers, which degrade more slowly in soil than unmodified flax when exposed in vitro to bacterial culture. For acetylation, the reaction was carried out at 30 °C for 3.5 h, and 0.4 vol% sulfuric acid was used as a catalyst. After 27 days of exposure, the remaining weight (%) was 28.1 ± 5.9 for acetylated fibers and 17.2 ± 4.6 for unmodified fibers. The acetylated fibers not only decompose slower than unmodified and PEG-grafted fibers, they also show a different morphology after the intervening exposure times of bacteria [9]. 

Rahman Bhuiyan et al. reported that treatment with chitosan can significantly increase the antibacterial activity of cotton fibers against Staphylococcus aureus and Escherichia coli [10]. 

Renouard et al. found that flax fiber yarns can be coated by dipping them in cellulose. The cellulose coating provides flax fiber yarns with complete physical protection that inhibits their degradation by the cellulase of a telluric microorganism. In addition, the presence of this coating provides the possibility of binding natural antibacterial compounds such as gramin or saponin, as the binding of an antibacterial phytoalexin that is effective against Gram-negative bacteria effectively prevented the development of cellulolytic bacteria such as Cellvibrio fulvus and Cellvibrio vulgaris, which were cultured in the presence of flax fiber yarns treated in this way to increase the lifetime of flax fiber-based geonets [11]. 

There is currently no commercial system that allows the targeted life extension of natural fibers while being fully biological, sustainable, and readily available. In this respect, the aim of this study was to investigate a sustainable solution to extend the service life of textiles made of natural fibers that are used in/on the soil and based on lignin.

Along with cellulose, lignin is a main component of wood and occurs in large quantities as a residue during paper production. As reviewed by Rosini et al., most of the industrial lignin is currently burnt for energy purposes. However, it is the richest natural source of aromatics and therefore a promising raw material for the production of value-added products [12]. 

According to Mili et al., lignin is promising for the production of biomaterials due to its richness, non-toxicity, and biodegradability. In their review paper, they mention numerous research papers that have confirmed the multifunctional applications of lignin, from wood adhesives in fiberboard, plywood, and particleboard, as well as binders in printed circuit boards and abrasive tools, to 3D printing, adhesive hydrogels, soil protectants, lignocellulosic paper, coatings, and others [13].

Lignin is already being researched for various applications in the textile industry. For example, it has been studied as a fire retardant and dyeing material for textiles [14]. It has also been referred to as being a potentially suitable UV blocker and as having antibacterial properties for linen fabric in the form of nanolignin [15]. 

Hult et al. investigated a lignin coating to improve the barrier properties of fiber-based packaging material. Commercially available softwood lignin was esterified with tall oil fatty acid (TOFA) and applied on the paperboard substrate. Then, the novel thermoplastic lignin samples were applied with a bar coater, forming an even coating on the substrate. In this way, the water vapor transmission rate of the paperboard (840 g/m^2^ × 24 h) could be reduced to 260 g/m^2^ × 24 h with a TOFA lignin ester double coating of 3.9 g/m^2^. Subsequently, the tensile strength of the coated paperboard was not affected [16].

Another study reported on the coating of coconut fibers, in which the lignin retards the oxidation and thermal degradation of the fibers in a polypropylene composite. The lignin used here was obtained using the Acetosolv process. By incorporating lignin into the composites, an increase in initial thermal decomposition temperatures and oxidation induction times was achieved [17].

Lignin from sugarcane bagasse was also applied on fabrics as a coating to be used as potential additional fabric in a hygiene mask. Sunthornvarabhas et al. reported on a lignin-coated fabric made from sugarcane bagasse that has antimicrobial characteristics against the bacterial strain Staphylococcus epidermidis (DMST 15505). In vitro studies showed that this bacterium can be inhibited at a specific coating concentration to prevent further spread within 6 h of contact with a lignin-coated, nonwoven sheet of glass fiber. They explain that the application of lignin on the antimicrobial textiles has the potential to replace silver nanoparticles; however, further development is needed to create a product that is attractive to users [18,19]. The studies indicate the potential of lignin in filtration and antimicrobial textile applications. Sirviö et al. and Upton et al. also reported on the suitability of the lignin material and its unique properties capable of developing films and coating materials, such as hydrophobicity, the ability to absorb UV light, and antioxidant and antimicrobial characteristics [20,21]. As also reported by Antunes et al., although lignin has interesting properties that can be explored for its use in the textile industry, further research in this area is needed to validate the potential and safety of lignin application [22].

Lignin is heterogeneous in composition and resistant to degradation, which significantly hinders its use in some applications [12]. However, for use as a protective coating of natural fiber geotextiles against biodegradation, this property can act as an advantage.

The unique natural structure of lignin, which is similar to the hindered phenols used as primary antioxidants in polymers, gives it its antioxidant properties [23,24,25]. Because of their wide effective temperature range (from ambient to processing temperatures), hindered phenols are widely used in geotextiles [26]. Such antioxidants continue to be effective even after disposal in landfills as they significantly slow down the decomposition process of the resulting polymer waste. The use of lignin as an antioxidant would therefore be of great benefit as it acts as an antioxidant throughout the life of the product and is still biodegradable [17].

Therefore, in this work, we coated cellulosic yarns with lignin with the goal of using them as an alternative to synthetic geotextiles by significantly extending the life of natural fiber materials. For this purpose, cotton yarn, the most used cellulose yarn worldwide, was coated with a hardwood-kraftwood lignin granulate using thermoplastic application methods. Soil burial tests were carried out with the coated yarns both in a climatic chamber and in an outdoor field. The degradation curves were established from tests of the loss of tensile strength over burying time. The loss of the tensile strength of the uncoated and lignin-coated yarns over burial time were compared as a function of coating thickness. In addition, two burial methods were compared with each other in order to determine the time acceleration due to defined increased humidity and temperature in the climate chamber and for extrapolation to calculate the life services correlating with the coating thickness.

## 2. Materials and Methods

**Figure 1:**
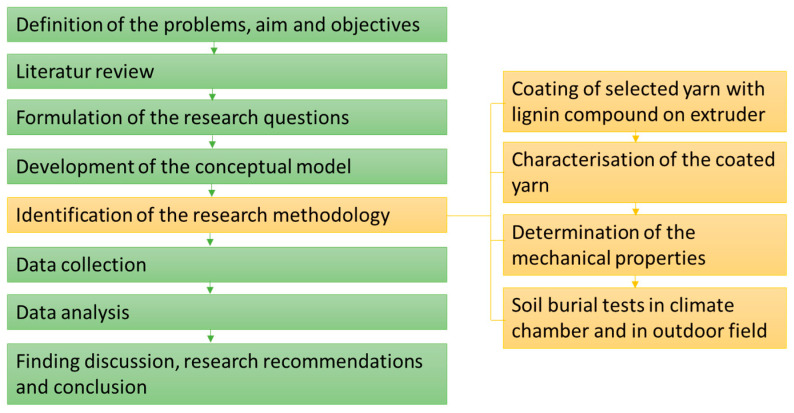
Overall flowchart for the research work.

To achieve the aforementioned objectives and to fill the identified research gap, a comprehensive research methodology comprising the phases in Figure 1 was adopted. After a thorough literature review on the degradation of natural fibers, biodegradable protective coatings for natural fibers, and lignin coatings for textiles, the research questions were formulated: “Which lignin and which reference yarn material should be used? How or with which equipment should the lignin be applied? Does the application thickness play an important role and which one? How should the degradation behavior be tested? Which property characteristics are most important, which pre- and post-tests have to be carried out? How should the results be evaluated?”. The working concept was then drawn up, and the methods were defined, both of which are explained in more detail in the following Section 2.2. Data collection was carried out concurrently, starting from the coating, the burial tests, and the optical and mechanical tests before and after burial. Collected data were analyzed with respect to, among other things, the maximum tensile strength loss. Two different burial methods, climate chamber and outdoor field, were compared. Thus, the outdoor tests were validated, and the time acceleration factor in the climatic chamber tests was calculated.

### 2.1. Materials

Yarn: The original yarn was a twisted yarn of 100% of cotton. This 5-ply yarn had a yarn count of Nm 14.

Lignin: The lignin-based thermoplastic used was a blend of hardwood-kraftwood lignin provided by Tecnaro GmbH, Ilsfeld, Germany. The granulate had a melt volume flow rate of 5 cm³/10 min (190 °C/ 2.16 kg acc. to ISO 1133 [27]), a heat distortion temperature of 41 °C (acc. to ISO 75 [28]), and an elastic modulus of 100 MPa (determined by 1 mm/min, according to ISO 527 [29]). 

*Soil*: According to RAL (quality assurance compost batch analysis), GZ 251 with test no.: 5117-174136-1 certificated compost, provided by Döbler GmbH, Kirchheim unter Teck, Germany, was used for the aging and burial tests so that the quality remained constant in order to achieve reproducible tests. This soil had a grain size of 0–10 mm, a density of 600 kg/m³, and dry matter of 64.8% with particularly high biological activity.

### 2.2. Methodology

First, the cotton yarn was coated with the lignin compound on the twin-screw extruder. Different coating thicknesses were applied to also determine the effect of the coating thickness on the degradation rate. The coated yarns were analyzed optically using a microscope and SEM as well as mechanically via tensile strength tests. The yarns characterized in this way and the original yarn samples were buried in the compost in both the climatic fern and field experimental fields. Experiments in the climatic chamber were carried out according to the given standards. Experiments in the outdoor field were designed and developed specifically for the project. Here, yarn samples were taken at regular intervals. All samples taken were characterized optically and mechanically and compared with the initial condition. Finally, the ratio obtained through climatic chamber and field trials was extrapolated as a time-lapse factor. The following section describes each method in more detail.

- Yarn coating

The cotton yarn was coated with lignin on a twin-screw extruder (PRISM EUROLAB 16 twin-screw extruder, Thermo Fisher Scientific Inc., Karlsruhe, Germany) with different coating nozzles for an even and adjustable coating layup around the yarn. Three sample variants were produced by varying the inner and outer diameter of the nozzle and/or the yarn coating speed. Table 1 provides an overview of the material samples produced and the extruder parameters:

b. Analysis of physical properties of the yarn samples

The coated yarns were characterized in terms of physical textile parameters. First of all, the coating thickness around the yarn was determined with the aid of optical methods (BX 53M Microscope, Olympus Europa SE & Co.KG Evident Europe GmbH, Hamburg, Germany). Further optical analyses on the yarn samples were performed using a scanning electron microscope (SEM) (TM1000 Tabletop Scanning Electron Microscope, Hitachi, Ltd., Tokyo, Japan).

The coating mass was determined by weighing the uncoated and coated samples with a laboratory scale (Mettler PM 200, Mettler Toledo GmbH, Gießen, Germany).

c. Analysis of mechanical properties of the yarn samples

**Figure 2:**
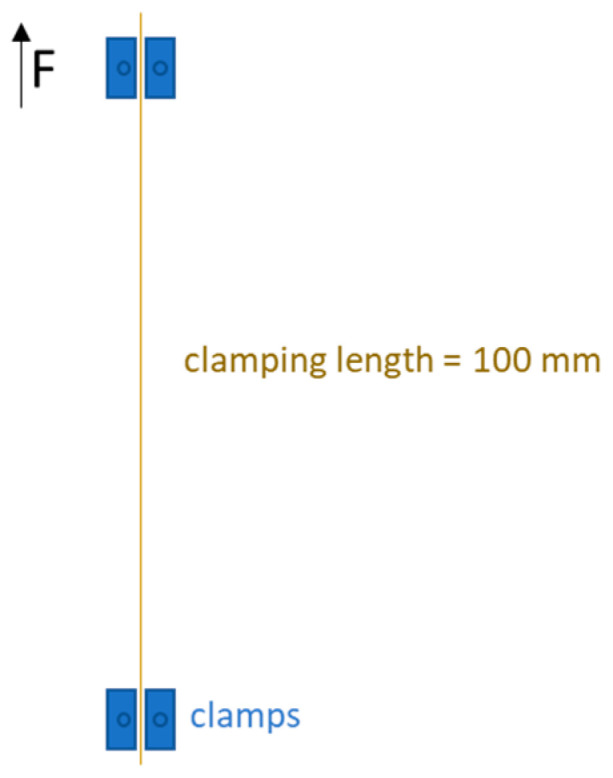
Schematic illustration and dimensions of a test specimen.

The mechanical properties of the uncoated and coated yarns are measured according to ISO 2062:2009 (Determination of single-end breaking force and elongation at break using constant rate of extension (CRE) tester) [30] as well as the titers according to DIN 53830-3:1981-05 (Testing of textiles; determination of linear density of single and plied yarns; simple yarns and plied yarns, textured yarns, short length method) [31] with the test device Zwick 1455, ZMART PRO (Zwick Roell, Ulm, Germany). Yarns are fixed between two ZZW Nr.22 (Vulkollan clamps) clamps (Figure 2). Tests were performed using a clamping length at start position of 100.00 mm, a measuring head of 100 N, and a preload of 0.5 cN/tex. The testing speed was 100 mm/min. At least 15 tests were performed for each sample.

d. Soil burial tests with the yarn samples

Soil burial tests in climate chamber: The uncoated and coated yarn samples were buried in direct contact with the soil according to standard ISO 11721-1:2001, Part 1 (Textiles—Determination of the resistance of cellulose-containing textiles to microorganisms; Soil burial test—Part 1: Proof of a rot-inhibiting finish) [32] and ISO 11721-2:2003 Part 2 (Determination of the resistance of cellulose-containing textiles to micro-organisms—Soil burial test—Part 2: Identification of long term resistance of a rot retardant finish) [33]. Certain lengths of yarn samples (min. 150 cm for *n* = 15 in the tensile tests) for each scheduled sampling over the burial period were formed into rolls and buried in soil into large clay plant pots (w × h × d = 16 × 15.5 × 43.5 cm) so that the individual samples were at least 2 cm apart from each other.

The water content of the test soil was set to 55–65% of the maximum moisture retention capacity and the pH of the test soil was in the range of 4.0 to 7.5. The beakers containing the buried samples were placed into the climatic chamber (Bacher Kälte—Klimatechnik GmbH, Waiblingen, Germany) for a burying period of 23 days. Incubation of the soil burial samples was carried out at 95 to 100% relative air humidity and 29 °C. Samples were systematically taken from the soil after 2, 5, 7, 12, 19, and 23 days of burial. After the defined burial time, the samples were removed from the soil and rinsed in ethanol/water (70/30 vol.%) solution for approximately 10 min before conditioning under textile testing in a laboratory climate at 20 °C/65 ± 2% RH); then, their mechanical properties were tested.

Soil burial tests in the outdoor field: The experiments were conducted from 12 May to 17 October 2022 at a site in Denkendorf, Germany (48°41′ N 9°20′ E). The uncoated and coated yarn samples were buried in direct contact with the soil in the field at a specially chosen site with sufficient solar radiation and rainfall. The same soil was used as that for the standard soil burial tests, and the samples were buried at a depth of approx. 16–17 cm. A total of 6 samples were taken over a 5-month burial period. The samples taken from the soil were treated with an ethanol/water (70/30 vol.%) solution for approximately 10 min before conditioning under textile testing laboratory conditions (20 ± 2 °C/65 ± 2% RH); then, their mechanical properties were tested.

## 3. Results

**Figure 3:**
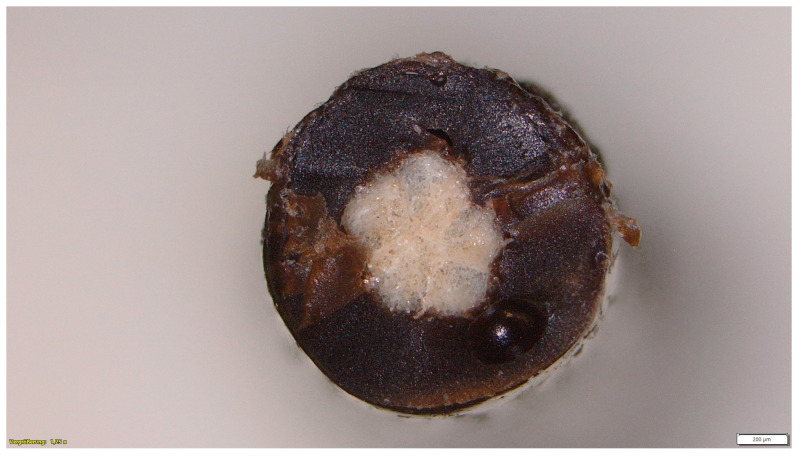
Microscopic image of yarn cross-section of coated sample 1 (magnification ×1.25; diameter of coated yarn approx. 946 µm).

Coating with lignin: Figure 3 shows the yarn cross-section sample 1. The microscope image proves that a uniform core-sheath coating has taken place.

The properties of the coated yarns are listed in Table 2.

The determined maximum tensile force (F_max_) and maximum tensile force elongation (e_max_) of the uncoated and coated yarns are shown in Table 3. As expected, the fiber thickness increased with the lignin coating. Moreover, the tensile force increased, resulting in a higher pick-up of the lignin coating due to the strength of the lignin film. Interestingly, the elongation increases slightly with the coating, which indicates an elastic behavior of the lignin film. 

### 3.1. Determination of the Residual Strength of the Yarn Samples Depending on the Burial Time

#### 3.1.1. Soil Burial Tests in Climate Chamber

The ISO 11721-1 standard specifies a method with which the resistance of the pretreated or coated textiles to the effects of the soil-present microorganisms is determined. Resistance to biodegradation is defined as the reduction rate in ultimate tensile force that occurs when the samples are buried in soil. This standard, therefore, gives a comparative statement between uncoated and coated yarns. It is less suitable for specifying soil resistance in absolute terms (i.e., days, months).

a. Resistance: The maximum tensile force values determined were plotted as a function of the burial time.

The soil burial test must continue until the control specimens have lost 80% of their ultimate tensile strength. For the tests with the yarns, the uncoated cotton yarn was used as a control sample. The duration of the soil burial process needed to reach this value was defined as time interval f_1_ and was expressed in days. The test samples, which were used to determine normal long-term stability, were taken from the soil when 2 × f_1_ was reached. The test samples, which were used to determine the increased long-term stability, were taken from the soil when 4 × f_1_ was reached:

f_2_ = 2 × f_1_ (1)

and

f_4_ = 4 × f_1_ (2)

where:

- f_1_: is the time interval in days needed until the control strips lose 80% of their maximum tensile strength; identification of a rot-retardant finish with no long-term resistance.
- f_2_: is the time interval in days needed to identify a rot-retardant finish with a regular long-term resistance.
- f_4_: is the time interval in days needed to identify a rot-retardant finish with an increased long-term resistance.

b. Residual strength: To calculate the residual strength, the maximum tensile forces of the coated samples were determined after the burial time f_1_ and set in relation to the respective maximum tensile force of the unburied sample:

q_f1 H,M_ = F_f1 H,E_/F_f1 H,O_ (3)

- F_f1 H,E_: maximum tensile force of the buried specimen after a burial time of f_1_.
- F_f1 H,O_: maximum tensile force of the unburied specimen.
- q_f1 H,M_: fraction of the remaining maximum tensile force after a burial time of f_1_.

If the reduction in maximum tensile strength is less than 25% (q_f1 H,M_ > 75%), the coating is considered to be rot retardant without considering long-term durability. 

c. Increased long-term stability: In the second part of the standard (DIN EN ISO 11721-2), the proportions of the remaining maximum tensile force are:

q_f2 H,M_ = F_f2 H,E_/F_f1 H,O_ (4)

And

q_f4 H,M_ = F_f4 H,E_/F_f1 H,O_ (5)

where:

- F_f2 H,E_: maximum tensile force of the buried specimen after a burial time of f_2_.
- q_f2 H,M_: fraction of the remaining maximum tensile force after a burial time of f_2_.
- F_f4 H,E_: maximum tensile force of the buried specimen after a burial time of f_4_.
- q_f4 H,M_: fraction of the remaining maximum tensile force after a burial time of f_4_.

If the reduction in maximum tensile strength after a burial time of f_2_ is less than 25% (q_f2 H,M_ > 75%), the effect of the coating is considered to be rot resistant with normal long-term durability. If the reduction in the maximum tensile force after a burial time of f_4_ is less than 25% (q_f4 H,M_ > 75%), the effect of the coating is considered to be rot resistant with increased long-term durability. 

**Figure 4:**
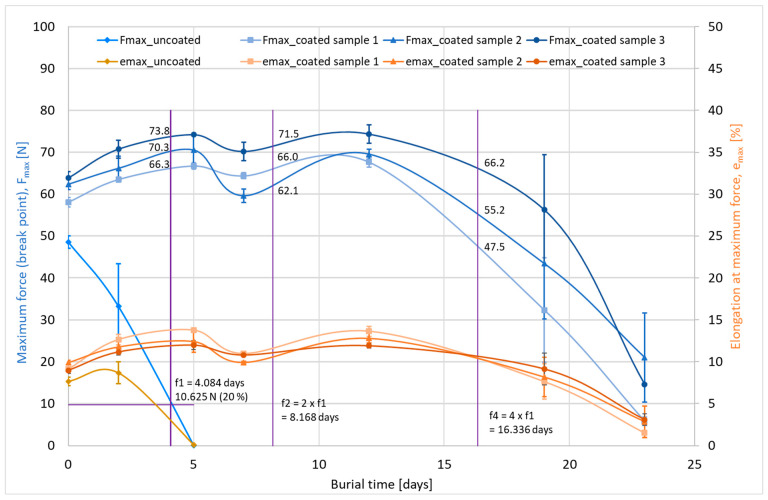
Change in mechanical properties of cotton yarns with and without lignin coating after burial in the ground according to DIN EN ISO 11721-1 and -2.

Figure 4 illustrates the results of the measurements on the coated cotton yarns. The uncoated cotton yarn was completely degraded after only approx. 5 days in soil in the climatic chamber, while the lignin-coated yarns showed a tensile strength reduction of approx. 70% at the end of a test period of approx. 23 days, as specified in the standard and depending on the coating thickness.

From this, the values of the maximum tensile forces for the burial times f_1_, f_2_, and f_4_ (4, 8, and 16 days) are examined for further calculation and classification (Table 4):

All q values (residual strengths) of the coated samples are greater than 75%. This means that these coated samples have a rot-retarding effect with long-term durability in accordance with DIN EN ISO 11721-1 and -2 compared to uncoated cotton yarn.

#### 3.1.2. Soil Burial Tests in Outdoor Field

The results from the outdoor field tests also confirm the longer service life of the lignin-coated yarn samples. The cotton yarn in its original condition (uncoated) was already severely attacked when the first sample was taken after 7 days. 

**Figure 5:**
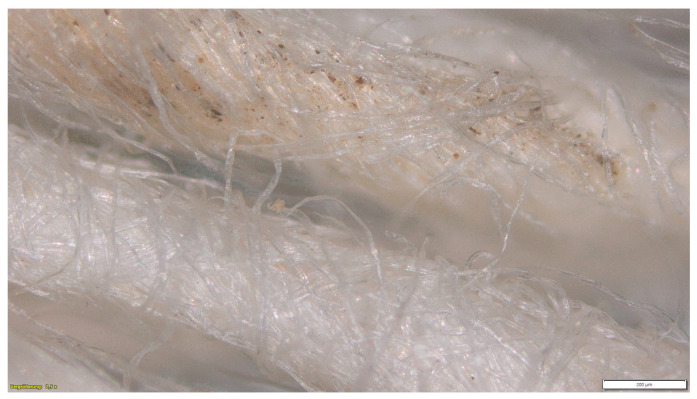
Microscope image of the uncoated yarn sample, showing the damaged fibers (magnification ×2.5).

Microscopic analyses on the uncoated yarn samples taken after a burial period of 7 days show the first damage on the fiber surface (Figure 5).

**Figure 6:**
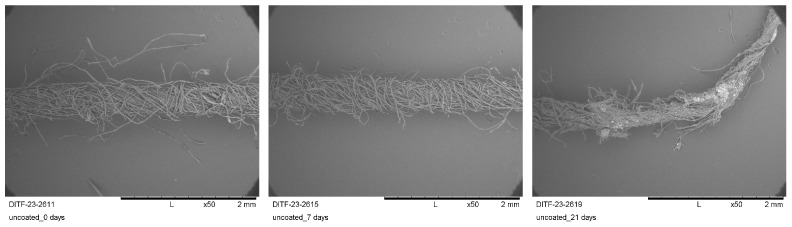
SEM images of the uncoated yarn sample, showing the damaged fibers (magnification ×50).

SEM images in Figure 6 show the fiber breaks after 7 and 21 burying days in the outdoor field compared to the unburied sample (burial time of 0 days, respectively).

The coated yarns, on the other hand, hardly showed any visual damage up to the end of the test period of 158 days. Even at the end of the entire burial period of 158 days in the outdoor test field, the coating was still intact—even with the thinnest coating. This is also confirmed by the SEM images of the coated sample 1, which were taken at different burial periods ranging from 0 to 158 days (Table 5).

**Figure 7:**
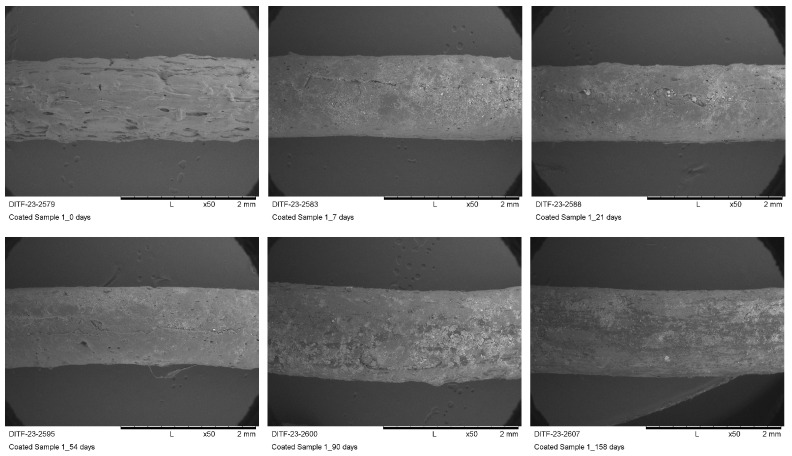
SEM images of the coated sample 1 after a burial period of 0 to 158 days (magnification ×50).

Longitudinal SEM images in Figure 7 show that the coating was still optical fully intact after 158 days of burial. 

**Figure 8:**
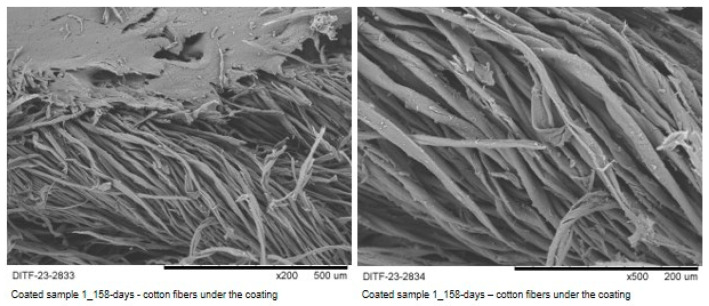
The cotton fibers in the coated sample 1 after a burying period of 158 days (left: part of the coating mass is visible) (magnification ×50).

Figure 8 shows cotton fibers in the coated sample 1 after 158 days of burial, after the top layer was cut open with a scalpel. Despite the long burying period, the fibers were fully protected against environmental influences by the lignin coating. 

**Figure 9:**
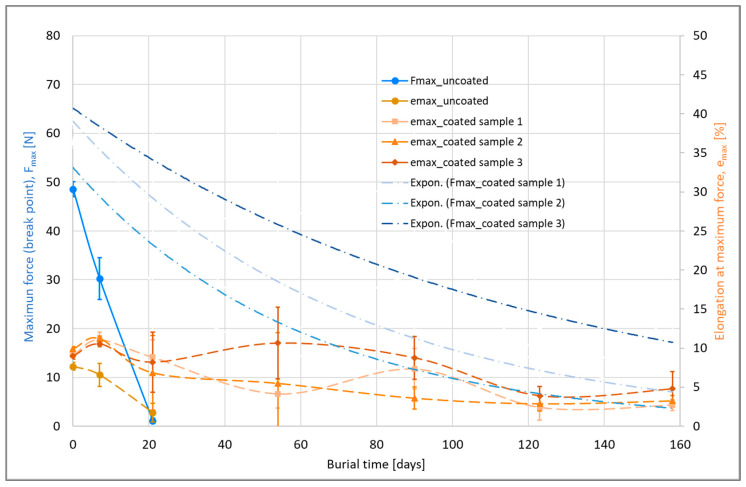
Reduction of the maximum tensile strength and the maximum elongation of the cotton yarns with and without a lignin coating from the soil burial tests in the outdoor field (Expon: exponential curve).

The uncoated yarn was completely degraded after 20 days, while the thinly coated yarn (coated sample 1) still had a maximum tensile strength of approx. 8 N after 158 days; moreover, the maximum tensile strength of the thickly coated yarn (coated sample 3) was approx. 20 N, with a maximum tensile elongation of 5% (Figure 9). The ultimate tensile strength also decreased for the coated yarns; however, the degradation rate reduced over time. 

### 3.2. Calculation of the Time Acceleration Factor in the Climate Chamber

The cotton yarns coated with lignin were buried both in the climatic chamber and in the field. In order to compare both methods with each other, and to calculate the time acceleration factor in the climatic chamber so that the absolute lifetimes for different coating thicknesses can be calculated, the following steps were taken:

1. We determined data points out of the burial tests and tensile strength tests for each yarn sample;
2. In order to compare the series of tests with each other, we placed best-fit curves through the data points. Exponential curves were ideal for this;
3. For each test series, we choose an exponential curve with a corresponding formula:
F_max_ = a × exp (b × t) (6)
  where:
  - F_max_ is the maximum tensile force;
  - t is the burial time in days;
  - a and b are parameters used to fit the curve to the data points.
4. The parameter reflects the start value (at t = 0) and b describes the course over time. Since it is a degradation, b is always negative;
5. For a specific sample, we had two series of data: one in the climatic chamber (cc) and one in the outdoor field (of); therefore, also had two exponential curves:
F_max_(cc) = a(cc) × exp (b(cc) × t) (7)
  and
F_max_(of) = a(of) × exp (b(of) × t) (8)
6. Degradation is faster in the climatic chamber, which explains why b(cc) < b(of) or |b(cc)| > |b(of)|;
7. The time acceleration factor can be calculated as b(cc)/b(of). It is independent of which maximum tensile force or degradation is considered.

**Figure 10:**
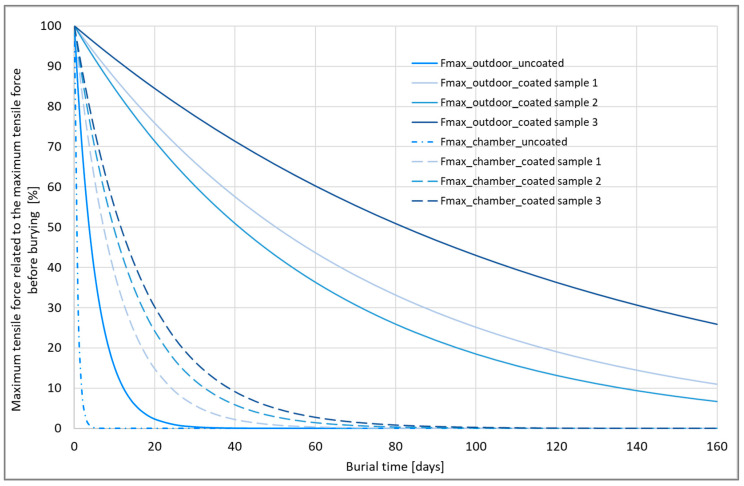
Comparison of the maximum tensile forces related to the maximum tensile force before burying in climate chamber and outdoor field.

Figure 10 compares the exponential degradation curves from the two tests after equating the starting points to 100%.

**Figure 11:**
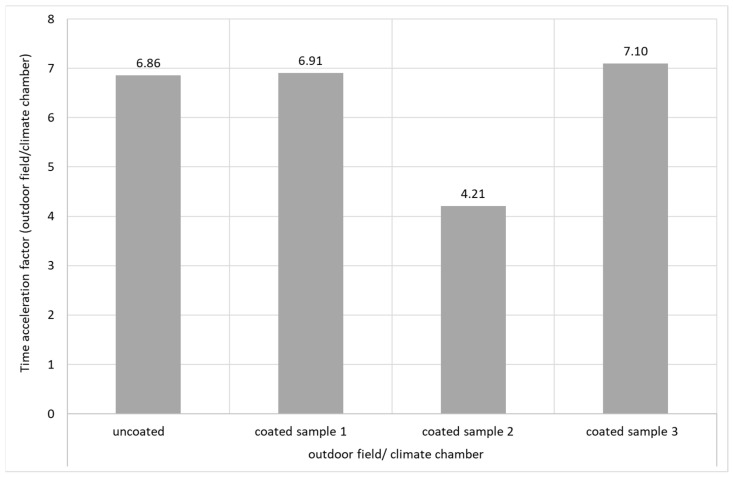
Time acceleration factor (acceleration of the biodegradation by treatment in the climate chamber).

The time acceleration factor between the climatic chamber and the field was calculated from these curves (Figure 11). This factor is approximately 7, with the exception of the coated yarn sample 2, which probably had defects caused by air bubbles in the lignin-coating layer, which are visible in the cross-section.

**Figure 12:**
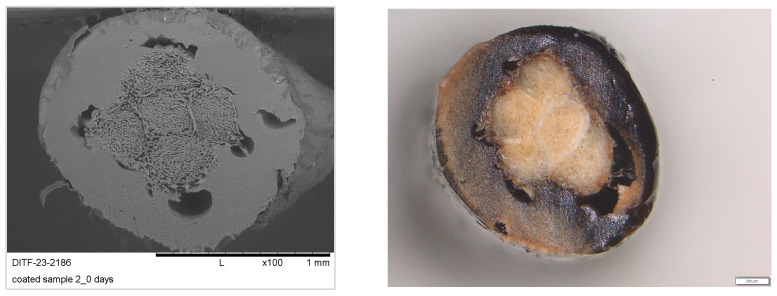
SEM and microscope images of the coated sample 2 in original condition and without burying (magnification ×100 and ×1.25, respectively).

Subsequent microscopic analyses on the coated samples showed that the sample had several air pockets in its cross-section (Figure 12), which can be explained by the residual moisture in the granules in front of extrusion coating. However, these air inclusions act as a weak point when tearing during the tensile test and thus provide incorrect information about the characteristic values determined. The values for the coated sample 2 are, therefore, probably outside of the expected range, between the coated samples 1 and 3.

## 4. Discussion

Compared to polysaccharides, the structure of lignin has far fewer polar groups, which means that lignins are hydrophobic and, therefore, insoluble in water and many other solvents. For this reason, they are more difficult to degrade biologically and chemically than other natural substances. In principle, lignins are decomposed to a limited extent by bacteria and, above all, fungi. For degradation, the fungi form thread-like hyphae that penetrate the lignin. Various enzymes are used to break down lignin, which are released into the medium by the fungus through exocytosis and diffuse into the lignin [34]. The major microbial degraders of lignin in our ecosystem are white-, brown- and soft-rot fungi and soil fungi. The effective biodegradation of lignin occurs through the generation of free radicals, which are derived from extracellular enzymes, such as laccases, lignin peroxidases, manganese-dependent peroxidase, dye-decolorizing peroxidase, and versatile peroxidase, that are produced by these fungi species [35].

However, due to their complex crosslinking, lignins are persistent natural substances and can only be decomposed very slowly by degraders. Lignin degradation always takes place under aerobic conditions and is very energy intensive. Accordingly, it cannot serve as the sole source of carbon and energy [36].

In this study, it has been shown that coating with lignin acts as a protective layer for natural fibers with a previously short lifespan in soil. For the uncoated cotton yarns, we observed similar results as in Arshad’s publication [2]: structural deformations were visible after the first week; moreover, after three weeks, the cotton fabric was so damaged that it was difficult to separate the remaining fiber pieces from the soil. This is also in agreement with the observations of Sülar and Devrim [4], where the cellulose fabric samples changed both physically and chemically after 1 month of burial, showing a high degradation, losing 90% of weight. The lignin-coated yarns in our study showed, on the other hand, hardly any damage at the end of the 158-day test period in the outdoor field.

In addition, the standard soil burial showed that the residual strength of the coated samples was greater than 75% for all three variations in the coating mass. This means that these coated samples have a rot-retarding effect with long-term durability compared to uncoated cotton yarn. This proves the high antibacterial effect of lignin, as shown in the studies of Morandim-Giannetti et al. [17] and Sunthornvarabhas et al. [19].

The achieved results indicate that the service life of natural textiles can be extended through a lignin-based coating of yarns. This increase in service life can be varied and adjusted by increasing the coating thickness. Another potential for extending the service life lies in the quality of the coating process technology. Microscopic images showed air pockets in the lignin layer. Due to the time-consuming tests in the field and the limited project duration, the work could not be repeated within the scope of the present study. After elimination through improved process control, a further increase in service life can be expected.

The compounding of the comparatively brittle lignin with more elastic biopolymers as well as the processing of the compounds on application systems for textiles requires the thermoplastic to be heated above the melting point. Appropriately, heatable systems and conveying devices for the melt are required for this. Compared to water-based recipes, a coating process using thermoplastic coating is less intensive regarding energy consumption, as no water has to be eliminated. 

## 5. Conclusions

More than 80 million tons of lignin are produced as a by-product of lignocellulosic biorefineries and the paper/pulp industry. These wastes also pose an environmental problem, as they are mostly burned as low-grade fuel, meaning a waste of natural resources [37]. On the other hand, due to the increasing scarcity of fossil resources and the environmental pollution caused by the use of synthetic materials, there is a constant search for new biomaterials. Therefore, for sustainable resource management, it is very important to increase the value of lignin utilization through new applications with technological improvements.

In this study, the waste product lignin—one of the most available raw materials on earth—has been shown to act as a protective layer for natural fibers with—until now—short lifespans in soil. The lignin-coated (core-sheath coating) cotton yarn was buried in a biologically active soil according to standards in a climatic chamber and in an outdoor field. It has been shown that the coated yarns have a significantly longer durability against the microorganisms in the soil compared to uncoated yarns. Long-term resilience has been demonstrated in standard burial tests. In the outdoor tests, the lignin coating was still fully intact even after about 160 days of burial. Visual observations and microscopic methods show that the core of the cotton yarn remained undamaged, with the uncoated yarn degrading completely after just a few days.

This study opens up new ways of using lignin to extend cellulosic fiber durability for soil applications, e.g., geotextiles.

Future research directions will cover the improvement of lignin compounding regarding further interesting properties for special textile applications. A process optimization will increase the protection effect and will extend the service time of textiles in soil.

## Figures and Tables

**Table 1:** Overview of the material samples produced and the corresponding extruder parameters.

Extruder Parameters	Coated Sample 1	Coated Sample 2	Coated Sample 3
Inside/outside nozzle diameter [mm]	1.0/1.4	1.0/1.4	1.0/2.0
Yarn coating speed [m/min]	10	5	5
Temperature in different zones (from inlet to outlet) [°C]	Zone 2 = 80; Zone 3 = 165; Zone 4 = 175; Zone 5 = 185; Zone 6 = 187; Zone 7 = 187		
Extrusion pressure [bar]	3		

**Table 2:** Coating properties of the coated yarns.

	Coated Sample 1	Coated Sample 2	Coated Sample 3
Average coating thickness [µm] (*n* = 5)	252	393	495
Coating mass [g/m of yarn] (*n* = 5)	0.864	1.266	2.128
Coating mass [% by weight] (*n* = 5)	227%	333%	560%

**Table 3:** Physical and mechanical properties of the coated yarns.

	Uncoated Sample	Coated Sample 1	Coated Sample 2	Coated Sample 3
fiber thickness [tex]	374.68 (s = 0.09)	1241.70	1649.50	2452.00
max. tensile force [N]	48.57 (s = 1.52)	58.10 (s = 1.11)	62.33 (s = 1.27)	63.85 (s = 1.48)
elongation at break [%]	7.65 (s = 0.49)	9.10 (s = 0.24)	9.90 (s = 0.29)	8.97 (s = 0.38)

**Table 4:** Maximum tensile strength and the loss of the maximum tensile strength of the buried and unburied specimens after the burial times f_1_, f_2_, and f_4_.

Sample of Yarn	F_f1 H,O_ [N]	F_f1 H,E_ [N]	q_f1 H,M_ [%]	F_f2 H,E_ [N]	q_f2 H,M_ [%]	F_f4 H,E_ [N]	q_f4 H,M_ [%]
uncoated	48.6	10.6	20.0	-	-	-	-
coated sample 1	58.1	66.3	114.1	66.0	113.6	47.5	81.8
coated sample 2	62.3	70.3	112.8	62.1	99.6	55.2	88.6
coated sample 3	63.8	73.8	115.6	71.5	112.0	66.2	103.7

**Table 5:** Pictures of uncoated and coated specimens after a burial period of 7 to 158 days.

	Burial Time in Outdoor Test Field [Days]					
	7	21	54	90	123	158
uncoated samples	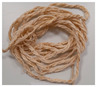	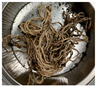	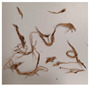	No residual materials, completely degraded.		
coated sample 1	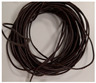	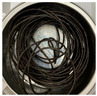	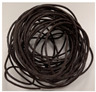	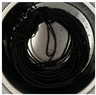	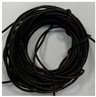	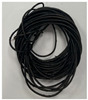

## Data Availability

Data sharing not applicable due to ongoing follow-up projects.
